# A bioisostere of Dimebon/Latrepirdine delays the onset and slows the progression of pathology in FUS transgenic mice

**DOI:** 10.1111/cns.13637

**Published:** 2021-03-23

**Authors:** Kirill Chaprov, Alexander Rezvykh, Sergei Funikov, Tamara A. Ivanova, Ekaterina A. Lysikova, Alexei V. Deykin, Michail S. Kukharsky, Alexey Yu. Aksinenko, Sergey O. Bachurin, Natalia Ninkina, Vladimir L. Buchman

**Affiliations:** ^1^ Institute of Physiologically Active Compounds Russian Academy of Science Chernogolovka Russia; ^2^ Engelhardt Institute of Molecular Biology Russian Academy of Sciences Moscow Russia; ^3^ Moscow Institute of Physics and Technology Dolgoprudny Russia; ^4^ Center for Precision Genome Editing and Genetic Technologies for Biomedicine Institute of Gene Biology Russian Academy of Sciences Moscow Russia; ^5^ Laboratory of Genome Editing for Veterinary and Biomedicine Belgorod State National Research University Belgorod region Russia; ^6^ Pirogov Russian National Research Medical University Moscow Russia; ^7^ School of Biosciences Cardiff University Cardiff UK

**Keywords:** ALS mouse model, drug effects, FUS, gamma‐carbolines, motor neuron disease, TLS

## Abstract

**Aims:**

To assess effects of DF402, a bioisostere of Dimebon/Latrepirdine, on the disease progression in the transgenic model of amyotrophic lateral sclerosis (ALS) caused by expression of pathogenic truncated form of human FUS protein.

**Methods:**

Mice received DF402 from the age of 42 days and the onset of clinical signs, the disease duration and animal lifespan were monitored for experimental and control animals, and multiple parameters of their gait were assessed throughout the pre‐symptomatic stage using CatWalk system followed by a bioinformatic analysis. RNA‐seq was used to compare the spinal cord transcriptomes of wild‐type, untreated, and DF402‐treated FUS transgenic mice.

**Results:**

DF402 delays the onset and slows the progression of pathology. We developed a CatWalk analysis protocol that allows detection of gait changes in FUS transgenic mice and the effect of DF402 on their gait already at early pre‐symptomatic stage. At this stage, a limited number of genes significantly change expression in transgenic mice and for 60% of these genes, DF402 treatment causes the reversion of the expression pattern.

**Conclusion:**

DF402 slows down the disease progression in the mouse model of ALS, which is consistent with previously reported neuroprotective properties of Dimebon and its other bioisosteres. These results suggest that these structures can be considered as lead compounds for further optimization to obtain novel medicines that might be used as components of complex ALS therapy.

## Graphical abstract

DF402, a fluorine derivative of Dimebon/Latrepirdine, delays the onset and slows the progression of pathology in the transgenic model of ALS‐FUS. An original CatWalk analysis protocol allowing detection of gait changes in FUS transgenic mice long before manifestation of clinical signs reveals effects of DF402 on their gait at early pre‐symptomatic stage. At this stage, DF402 also reverts expression of many genes whose expression in the spinal cord of FUS transgenic mice is already affected.

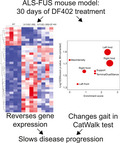

## 1 INTRODUCTION

Amyotrophic lateral sclerosis (ALS) is a neurodegenerative disease primarily characterized by dysfunction and death of lower and upper motor neurons. There is no cure for this fatal disease, and the efficacy of Riluzole and Edaravone, the only drugs approved for treatment of ALS patients, is very low. Suggested mechanisms of action for these two drugs are different; Riluzole is believed to be neuroprotective because of its ability to block glutamate excitotoxicity, whereas Edaravone acts as a scavenger of oxygen radicals (reviewed in Refs^1^, ^2^). A growing number of re‐purposed and new drugs as well as alternative therapeutic approaches entering clinical trials (for recent review, see Refs^3^, ^4^) raise hope that efficient treatment of different forms of familial and sporadic ALS could be achieved by designing tailored combinations of traditional pharmacological and modern biomedical approaches. Therefore, a search for potential components of efficient ALS therapy should include various types of potentially neuroprotective drugs, particularly those with pleiotropic effects on physiological and pathological processes in the nervous system.

In the last decade, gamma‐carbolines have attracted attention as potential neuroprotectors in the nervous system affected by neurodegenerative processes.^5^, ^6^, ^7^, ^8^, ^9^, ^10^, ^11^, ^12^, ^13^, ^14^ Their neuroprotective activity can be explained by a combination of multiple mechanisms of action described for these compounds.^11^, ^28^ Irreversible pathological aggregation of certain proteins contributes to pathogenesis of all types of ALS and therefore, it is an obvious target for therapeutic intervention. The first gamma‐carboline found to affect pathological protein aggregation was Dimebon (also known as Latrepirdine), originally approved and for many years used as an antihistamine drug. An ability of Dimebon to prevent accumulation of cytoplasmic proteinaceous inclusions has been first demonstrated in cultured neurons expressing a highly aggregation‐prone variant of RNA‐binding protein TDP‐43.^29^ Further studies in various cellular models of proteinopathies confirmed anti‐aggregation properties of Dimebon and demonstrated its ability to activate autophagic mechanism of pathological aggregate elimination.^10^, ^11^, ^21^, ^22^, ^27^, ^30^ Consistently, chronic treatment with Dimebon, when started at a pre‐symptomatic stage of the disease, delayed the onset of clinical signs, slowed down disease progression, and increased animal lifespan in several mouse models of neurodegeneration, including those that recapitulate key features of ALS.^7^, ^9^, ^28^, ^31^, ^32^ However, the efficacy of Dimebon in ameliorating pathology in these models was low and in attempt to produce more efficient compounds, several bioisosteres of Dimebon have been synthesized. Neuroprotective properties have been demonstrated for some of fluorinated bioisosteres in mouse models.^9^, ^12^

Here, we studied effects of one of these bioisosteres, DF402, on the disease triggered in transgenic mice by neuron‐specific expression of a highly aggregation‐prone truncated form of human FUS.^33^

## 2 MATERIALS AND METHODS

### 2.1 Animals

Transgenic mice expressing C‐terminally truncated form of human FUS under control of the neurospecific Thy‐1 regulatory sequences (FUS[1–359]‐line 6) have been produced and characterized previously.^33^ A line S‐FUS[1–359] produced by backcrossing of the original FUS[1–359] line 6 mice with CD1 mice^34^, ^35^ was used in the current study. Cohorts of experimental animals were formed from hemizygous transgenic and wild‐type male littermates. Genotyping was carried out using a PCR protocol described previously.^33^ Animals were housed in a specific pathogen‐free facility with controlled environment (temperature 21 ± 2°C, a humidity of 40%–60%, 12‐h light/dark cycle) and ad libitum access to food and water. Animal work was carried out in accordance with the ARRIVE guidelines 2.0^36^ and the United Kingdom Animals (Scientific Procedures) Act (1986) and was approved by The Bioethics committee of Institute of Physiologically Active Compounds, Russian Academy of Sciences (Approval No. 20 from 23.06.2017).

### 2.2 DF402 treatment and monitoring of the disease phenotype

Dimebon bioisostere DF402 (2,8‐Dimethyl‐5‐[2‐(6‐trifluoromethylpyridin‐3‐yl)ethyl]‐2,3,4,5‐tetrahydro‐1*H*‐pyrido[4,3‐*b*]indole dihydrochloride) was synthesized in IPAC RAS as described elsewhere.^37^ Similar to DF302, another fluorine‐containing and structurally close derivate of Dimebon, that was previously assessed for its ability to ameliorate neurodegenerative processes in various models,^9^, ^12^ DF402 is water soluble, stable in aqueous solutions, and non‐toxic to cultured cells and experimental animals. Animals received DF402 in the drinking water (70 µg/ml; correspondent to daily dose of 12 mg/kg/day after adjustment to average daily water intake in these mice) from the age of 42 days. The same administration protocol and a drug dose were used for assessing the efficacy of Dimebon in our previous studies^9^, ^31^, ^38^ and in two mouse models of neurodegeneration, a slowing down of pathology progression was achieved.^9^, ^31^ To collect data about the disease onset, disease duration, and animal lifespan, mice were assessed daily for the development of first signs of neuronal pathology (paresis of a limb, unstable gait, hunched posture, clasping reflex, decreased motility). Observations continued after the disease onset and animals were humanely killed when their conditions reached the severe level as specified by the Home Office Licence.

### 2.3 Animal gait analysis

CatWalk XT system Version 10.6 was used according to the manufacturer (Noldus Information Technology, Netherlands) instructions with the Intensity of the Green Walkway Light 16,5 V; Camera Gain 20 dB; Green Intensity Threshold 0,1; and Red Ceiling Light 17,7 V. All animals had one habituation session to the CatWalk apparatus one day before the first test. Each animal was tested every or every second day until the onset of clinical signs. On the testing day, each animal was allowed to explore and walk freely through the apparatus detection “runway” without any rewards. During this test period of maximum 10 minutes, at least 3 videos of an uninterrupted crossing of the recording field of the runway (approximately 35 cm) were automatically recorded. Runs for analysis were selected based on a minimum of four step cycles in the crossing field, such that each step cycle involved capturing the use of each of the four paws irrespective of the order in which paws were used. After classification of the footprints in these runs using the CatWalk software, qualified data^39^, ^40^, ^41^ were exported for further analysis to the RStudio.^42^ Given that the disease progression has individual characteristics resulting in a floating date of the manifestation of clinical signs, we have compared changes in the gait of animals in the reverse direction starting from the day when first clinical signs were observed to the day of the beginning of data collection. In order to combine the data of gait analysis between the animals with different ages, time intervals were adjusted to 25 days for each animal starting from the day of the manifestation of clinical signs, as illustrated in Figure S1. Statistical analysis was done using nonparametric Wilcoxon test for each of CatWalk parameters of untreated and DF402‐treated mice. The difference was considered significant if it was determined in at least one of the time intervals: 1–10 days, 11–18 days, and 19–25 days with *p* < 0.05 (Benjamini‐Hochberg correction). Representative analysis of significantly changed group of variables was performed by calculating the frequency of components implemented in the intrinsic structure of CatWalk parameters. For example, parameter “Right_Hind_MaxIntensity” consists of two components “Right_hind” and “MaxIntensity” representing qualitative and quantitative indicators. The frequency was determined as a ratio of the significantly changed parameters or components included in such parameter (*p* < 0.05; Benjamini‐Hochberg correction) to all CatWalk parameters. For principal component analysis, the values of all parameters were transformed to *z*‐scores ((*x*‐mean(*x*)/sd(*x*)). The multivariate analysis of variance^43^ was used to find traits with significant changes between experimental groups. Classical (Torgerson) multidimensional scaling (MDS)^44^ was performed to estimate clusterization between mice groups. Visualization was performed using custom scripts written in R.

### 2.4 RNA sequencing

For RNA‐seq analysis, total RNA was extracted from the thoracic and lumbar spinal cords of experimental and control pre‐symptomatic mice (70‐day‐old, 4 animals per group) using Qiagen RNeasy Plus mini kit. RNA quantification, quality controls, and further steps were performed as described previously.^34^ Briefly, cDNA libraries for the dual indexed single‐end sequence analysis were prepared from equal amounts (270 ng) of each total RNA sample using Illumina TruSeq Stranded Total RNA LT Sample Prep Kit. Following quality checks and normalization, cDNA libraries were sequenced on Illumina NextSeq 500 to generate single‐end 75 bp reads.

Raw sequence data processing (QC, trimming, alignment, read quantification) was performed with PPLine tool.^45^ Differential gene expression analysis was performed with the edgeR package.^46^ Gene Ontology and KEGG enrichment analyses were performed using topGO (v.2.36.0) and clusterProfiler packages.^47^ Multidimensional scaling (MDS) between all experimental samples was performed with limma package.^48^

Sorting of microglial and neuronal genes of the whole spinal cord samples was performed as described previously.^34^, ^49^

Compensation coefficient of DF‐402 treatment on FUS‐mediated transcriptomic changes was calculated as a percentage of [mean(logCPM)S‐FUS[1–359] — mean(logCPM)S‐FUS[1–359]+DF402)] / [mean(logCPM)S‐FUS[1–359] — mean(logCPM)WT]. Density distribution of compensated genes was estimated using Kernel Density Estimation (KDE).

Visualization of gene set enrichment analysis (GSEA) was performed using custom scripts written in Python and R. Raw data of RNA‐seq for untreated and DF402‐treated S‐FUS[1–359] mice were deposited in NCBI GEO database under the number GSE161680. RNA‐seq data of wild‐type animals were deposited previously by the number GSE130604.

The real‐time RT‐qPCR analysis of mRNA expression was carried out as described previously.^34^, ^50^

### 2.5 General statistical analysis

Data sets for the disease onset, disease duration, animal lifespan, and RT‐qPCR analysis of RNA expression were assessed for normal distribution and for those that passed D'Agostino and Pearson test, statistical significance of observed difference was evaluated by one‐way ANOVA or paired *t*‐test, as appropriate. For not normally distributed data, Kruskal‐Wallis ANOVA and/or Mann‐Whitney U‐test were used.

## 3 RESULTS

### 3.1 Chronic treatment with DF402 delays onset and prolongs duration of the disease in transgenic mice expressing C‐terminally truncated form of human FUS

**FIGURE 1:**
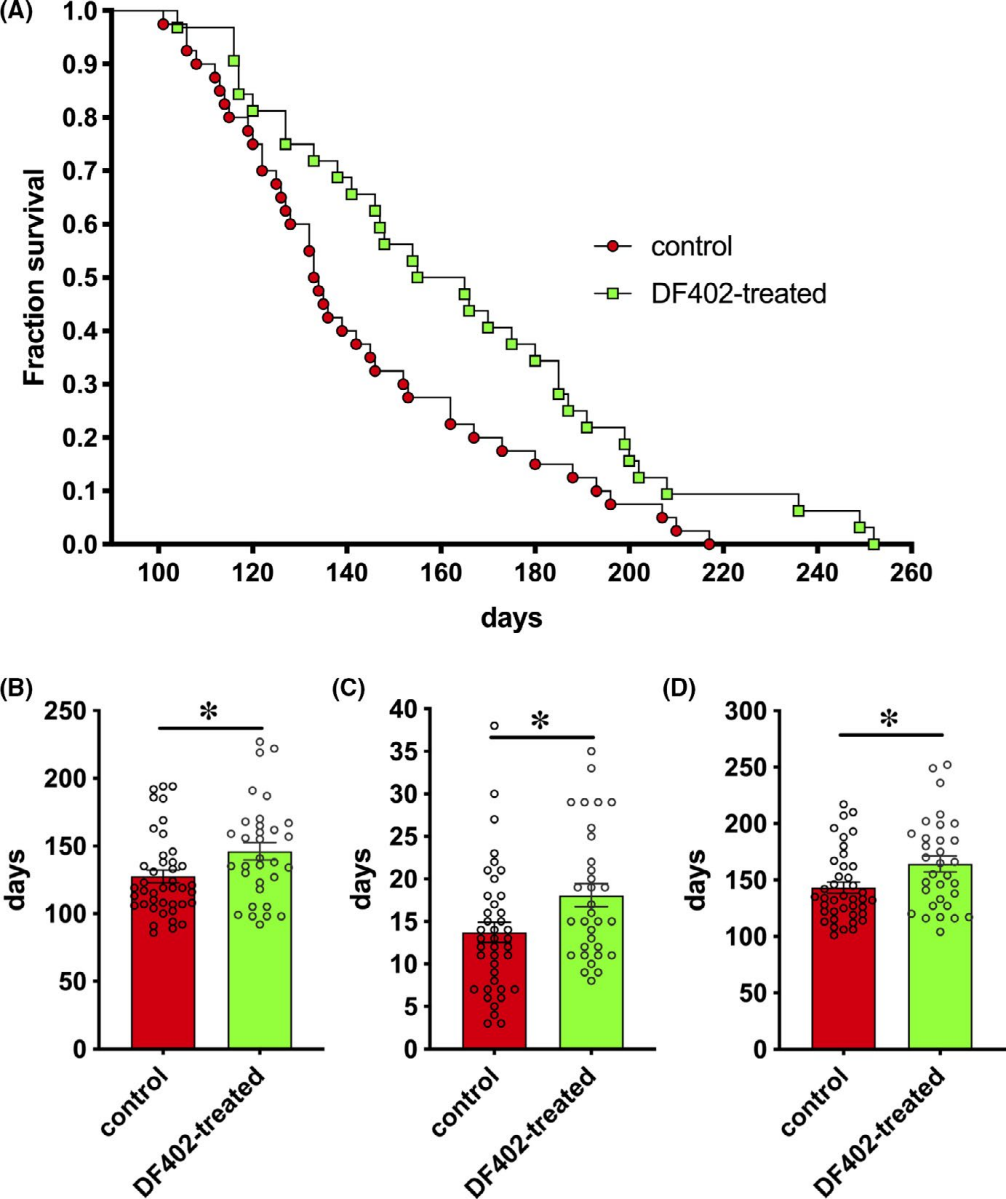
Effect of DF402 on the disease parameters in S‐FUS[1–359] transgenic mice. Experimental group of hemizygous FUS transgenic mice received DF402 in drinking water (70 μg/ml) from the age of 42 days. Kaplan‐Meier plot of animal survival in cohorts of hemizygous control (red circles, *n* = 40) and DF402‐treated (green squares, *n* = 32) littermate FUS transgenic mice. Log‐rank test revealed significant difference between survival curves, *p* = 0.0214 (A). Bar charts show means ± SEM and unpaired t‐test for the disease onset (B, *p = 0.0371), disease duration (C, *p = 0.0175), and animal lifespan (D, *p = 0.0146) in cohorts of control (*n* = 40) and DF402‐treated (*n* = 32) mice

Male S‐FUS[1–359] transgenic mice received DF402, a bioisostere of Dimebon, as described in Materials and Methods. The treatment started soon after weaning, from 42 days of age, which allows at least 7 weeks of drug administration before the earliest onset of pathology observed for this transgenic line. Animals treated with DF402 and their control male littermates were checked daily for the presence of discernible clinical signs of the disease. The age of the disease onset denoted by the first manifestation of clinical signs was recorded, and monitoring of animal health was continued until their conditions deteriorated to the level when they were deemed to be premorbid and therefore were euthanized by a Schedule 1 method. We found that chronic DF402 treatment increases animal lifespan by 13% (Figure 1A,D), which was due to both a later onset (Figure 1B) and longer duration (Figure 1C) of the disease (by 11% and 24%, respectively).

### 3.2 CatWalk analysis of the gait of transgenic mice expressing C‐terminally truncated form of human FUS detects impairments of motor function and effects of DF402 at pre‐symptomatic stage

**FIGURE 2:**
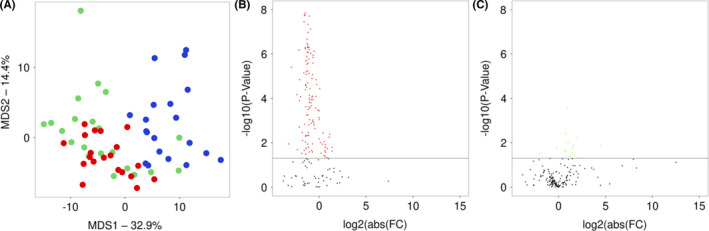
Clusterization of experimental mice at the age of 70 days using CatWalk data analysis and sorting of statistical differences in changes of gate parameters. (A) Results of classical (Torgerson) MDS analysis are shown. Each dot represents an experimental animal: blue for WT mice, red for untreated S‐FUS[1–359] mice, and green for DF402‐treated S‐FUS[1–359] mice. Total fraction of variance explained by principal components 1 (x‐axis) and 2 (y‐axis) was 47.3%. (B) Volcano plot illustrates the number of parameters that display significant changes between WT and S‐FUS[1–359] mice. Black line on y‐axis represents threshold of significant changes of parameters (−log10(0.05)). Red and black dots represent parameters with and without statistically significant changes, respectively. (C) Volcano plot illustrates the number of parameters that display significant changes between untreated and DF402‐treated S‐FUS[1–359] mice. Black line on y‐axis represents threshold of significant changes of parameters (−log10(0.05)). Green and black dots represent parameters with and without statistically significant changes, respectively

To assess whether instrumental analysis of multiple parameters of animal gate would allow detecting the decline of animal motor performance long before obvious manifestation of clinical signs and revealing any improvements of this performance as the result of DF402 treatment, we used CatWalk XT system to systematically monitor gait of DF402‐treated and control, untreated S‐FUS[1–359] transgenic mice as well as their wild‐type littermates. For this part of the study, additional cohorts of 24 wild‐type and 49 S‐FUS[1–359] transgenic male littermate mice were produced. The same as above protocol of DF402 administration was used to treat 25 of these S‐FUS[1–359] mice from the age of 42 days. Four weeks later, that is, at the age of 70 days, four animals from each group were humanely euthanized and tissue samples were collected for transcriptomic analysis (see below). It is important to note that at this age, all transgenic animals were undistinguishable from their wild‐type littermates and in this cohort of S‐FUS[1–359] mice, a mean age of the disease onset was 124 days for untreated and 137 days for DF402‐treated animals. However, the CatWalk analysis of 177 gait parameters carried out as described in Materials and Methods revealed differences between groups already at the age of 70 days: 128 parameters were found different between untreated S‐FUS[1–359] and wild‐type mice and 16 parameters—between untreated and DF402‐treated S‐FUS[1–359] mice (Figure 2). The latter two groups of mice have continued to be regularly assessed on the CatWalk until the first day of the disease onset denoted by the manifestation of clinical signs. Because the timing of the disease onset varies substantially between individual S‐FUS[1–359] mice, we analyzed changes in their gait in the reverse direction from the actual day of the onset as illustrated in Figure S1. We considered the time interval of 25 days as the pre‐symptomatic stage for all of the studied animals. This pre‐symptomatic stage was subdivided into three dynamic ranges: 1) 25–15 days (early stage), 2) 14–8 days (middle stage), and 3) 7–1 day (late stage) to the day when discernible clinical signs were first detected.

**FIGURE 3:**
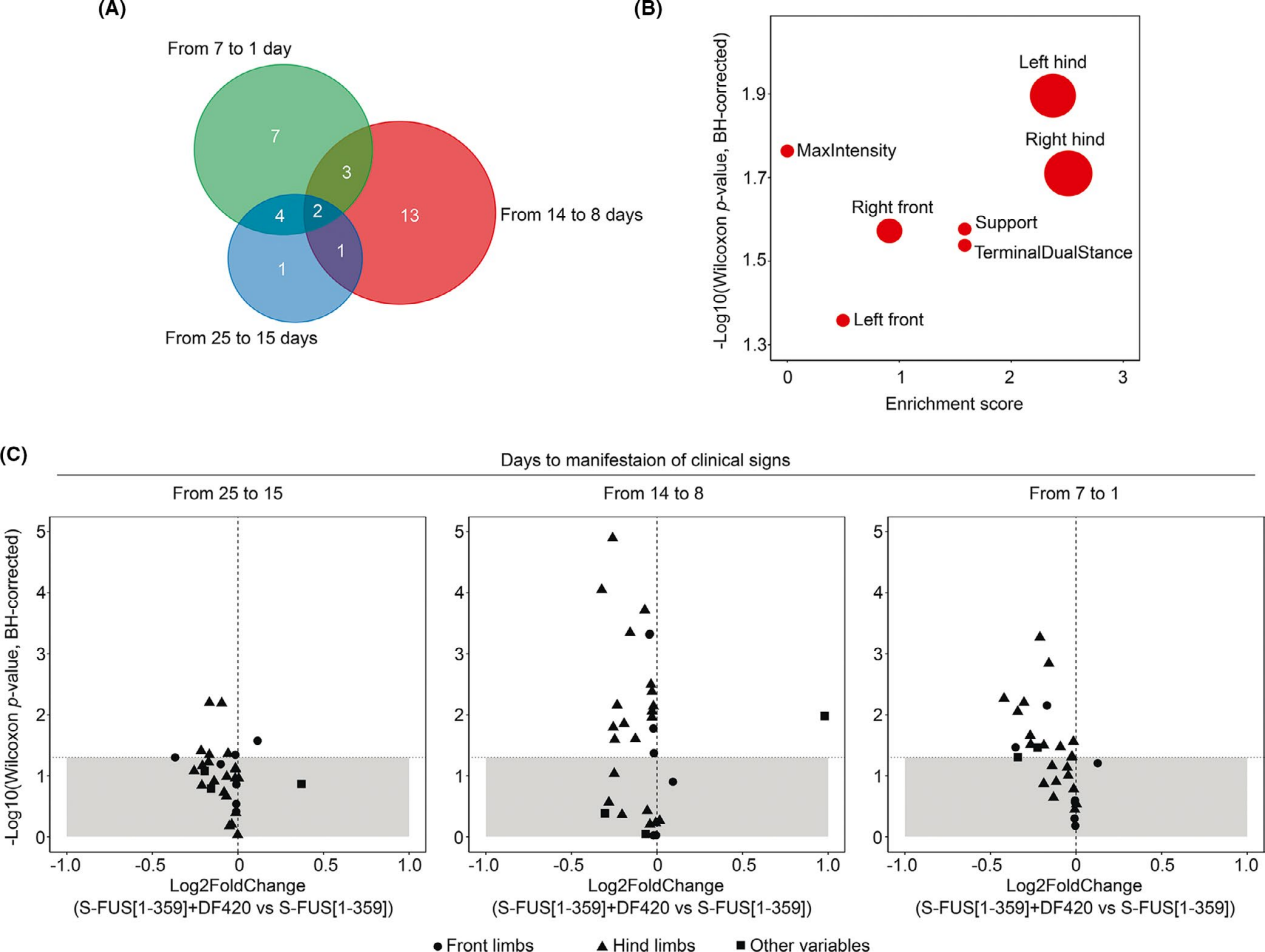
Comparative analysis of the gait changes at the pre‐symptomatic stage between untreated and DF402‐treated S‐FUS[1–359] mice. CatWalk XT system was used to measure gait parameters during the pre‐symptomatic stage subdivided into three intervals (from 25 to 15, 14 to 8, and 7 to 1 days) as explained in the Material and Methods and Results sections and illustrated in Figure S1. (A) The Venn diagram shows the number of gait parameters that display statistically significant changes at three substages of the pre‐symptomatic stage as the result of DF402 treatment. (B) The graph illustrates frequency of occurrence of variables exhibiting significant changes for the studied transgenic animals at any of three intervals of the pre‐symptomatic stage. The size of each bubble indicates a number of variables for a particular qualitative and quantitative indicator, for example, limb or intensity. Enrichment score was calculated following equation log2(freq_significant / freq_overall), where freq_significant—frequency of the parameter among the list of significantly changed parameters, and freq_overall—frequency of the parameter among the general list of the parameters. Parameters with enrichment score lower than 0 were discarded. (C) Log2 fold change of gait parameters between untreated and DF402‐treated S‐FUS[1–359] mice separately for early, middle, and late intervals of the pre‐symptomatic stage. Gray zone represents insignificant changes

The comparative analysis of the gait between DF402‐treated and untreated S‐FUS[1–359] transgenic mice revealed 31 parameters that display significant changes at one, two, or all three substages of the pre‐symptomatic stage: 8 parameters at the early stage, 19 at the middle stage, and 16 at the late pre‐symptomatic stage (Figure 3A). Most of the parameters affected by the DF402 treatment at the pre‐symptomatic stage attribute to the indicators of front and hind limb movements, including stride length and width, and the intensity of paws pressure rather than indicators of the general body movements, like speed or running cadence (Figure 3B). We also noted that the values for most gait parameters that displayed statistically significant changes appeared to be decreased after the DF402 treatment (log2 fold change from −0.5 to −0.05, *p* < 0.05, Wilcoxon test, BH corrected) (Figure 3C).

### 3.3 Expression of a number of genes in the spinal cords of transgenic mice expressing C‐terminally truncated form of human FUS is already changed at pre‐symptomatic stage and treatment with DF402 reverts expression of more than a half of these genes

In an attempt to reveal molecular processes and mechanisms that are affected by chronic DF402 treatment and might be involved in the delay of the disease onset in S‐FUS[1–359] transgenic mice, we compared spinal cord transcriptomes of 70‐day‐old untreated and DF402‐treated transgenic mice. At this pre‐symptomatic stage, no neurodegenerative changes (eg, neuronal loss or neuroinflammation) that significantly affect expression pattern at the symptomatic stage are present in the spinal cord of S‐FUS[1–359] mice.^33^ However, certain functional changes can be already detected at this age by comparing CatWalk gait analysis data for transgenic and wild‐type littermate mice (Figure 2A,B); moreover, such analysis can reveal differences between untreated and DF402‐treated mice (Figure 2C).

cDNA libraries for RNA‐seq were prepared from the spinal cord samples of four animals per genotype/treatment group and as a result of deep sequencing, we obtained ~15 million reads for each library. Differential gene expression analysis revealed relatively modest changes (37 differentially expressed genes (DEG) with LogCPM >2, FDR <0.05) in the transcriptome of S‐FUS[1–359] mice compared to the transcriptome of age‐matched wild‐type littermates, while transcriptome of DF402‐treated S‐FUS[1–359] mice showed more such changes (171 DEG with LogCPM >2, FDR <0.05) and can be clearly distinguished on multidimensional scaling (Figure S2A, Table S1). To discriminate neuron‐specific and microglia‐specific genes among the identified DEG, we used previously published data sets of purified microglia and laser‐microdissected ventral horns of the mouse spinal cord.^51^, ^52^ Between all identified DEG, 64% belonged to genes with exclusive or predominant expression in neurons and only 6%—to genes with expression intrinsic to microglial cells of the mouse spinal cord, while other 30% of DEG were common for both types of cells and were considered as shared (Figure S2B). Thus, at the pre‐symptomatic stage the observed gene expression changes in the spinal cord take place predominantly in neurons.

**FIGURE 4:**
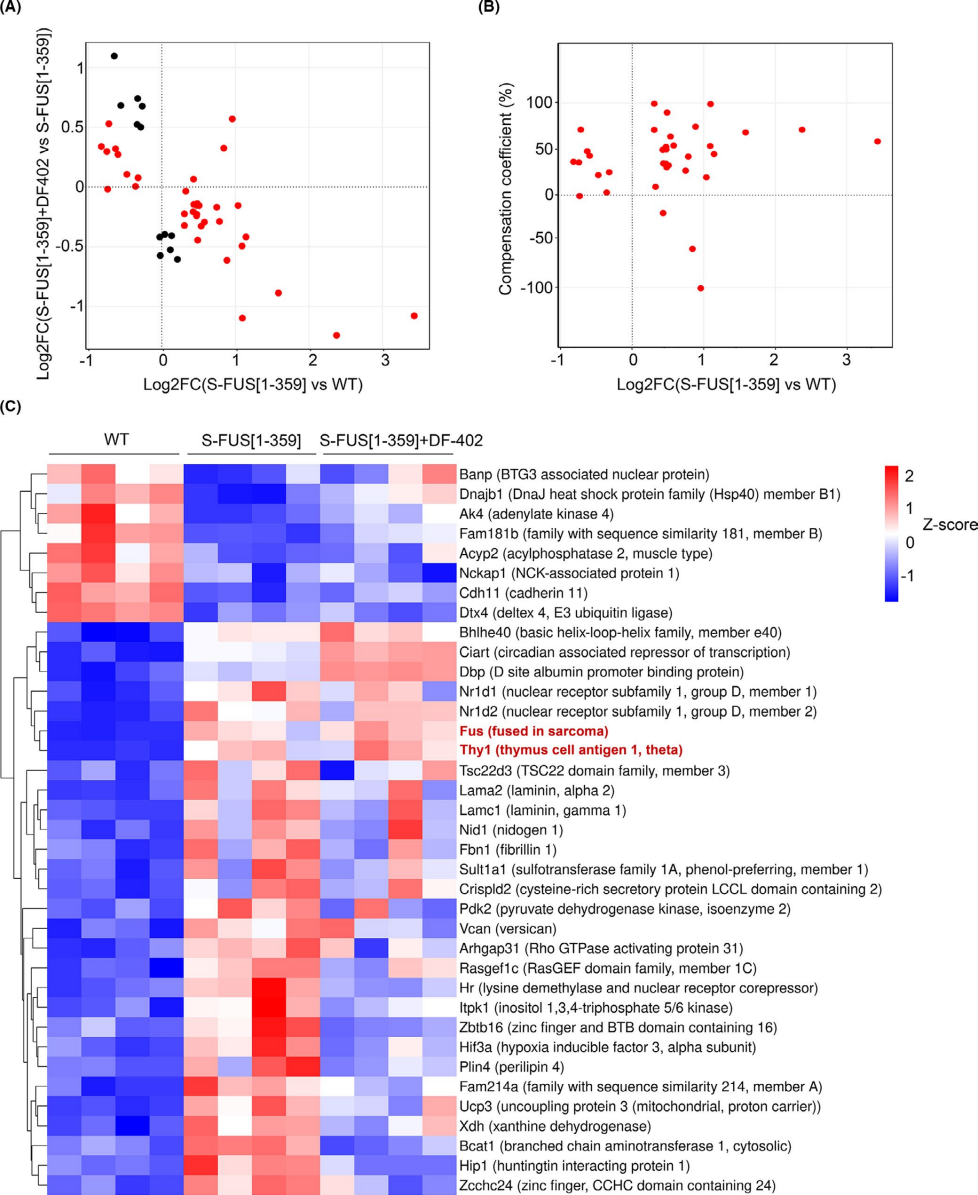
Compensation of FUS‐mediated transcriptomic changes by DF402 treatment. (A) Comparison of log2 fold change for genes that show difference in expression levels between S‐FUS[1–359] and WT transcriptomes (x‐axis), and between S‐FUS[1–359] and S‐FUS[1–359]+DF402 transcriptomes (y‐axis). Positive values correspond to activation in transgenic mice or under treatment. One point represents one gene, and only genes exhibiting significant changes (FDR <0.05) are shown. Genes that display difference between S‐FUS[1–359] and WT transcriptomes are shown in red, and those that display difference only between S‐FUS[1–359]+DF‐402 and S‐FUS[1–359] transcriptomes—in black. (B) Dependence of compensation coefficient (y‐axis) on log2 fold change in the S‐FUS[1–359] vs WT comparison (x‐axis). Compensation coefficient was calculated as a percentage of [mean(logCPM)S‐FUS[1–359] — mean(logCPM)S‐FUS[1–359]+DF402)] / [mean(logCPM)S‐FUS[1–359] — mean(logCPM)WT]. (C) Heatmap of differentially expressed genes between groups S‐FUS[1–359] vs WT and S‐FUS[1–359] vs S‐FUS[1–359]+DF‐402. The expression values of the genes are Z‐transformed. Products of the transgene cassette expression, Fus and Thy1, that were not used in analysis of compensation shown in panels a and b, are still included in the heatmap with gene names shown in red

When changes in gene expression between S‐FUS[1–359] and wild‐type groups were compared with changes in gene expression between S‐FUS[1–359] and DF402‐treated S‐FUS[1–359] groups, we found negative correlations of log2 fold changes (Spearman correlation = −0.55, *p* < 0.05) with over 80% of DEG exhibit opposite directions of expression changes (Figure 4A). Such pattern of gene expression changes might indicate that DF402 administration alleviates the effect of FUS transgene expression in the motor neurons. To quantify the compensation effect mediated by DF‐402 treatment, we calculated the compensation coefficient as described in the Materials and Methods section. According to the formula used for calculations, the coefficient near 100% means almost complete compensation by treatment, higher values correspond to overcompensation and negative values correspond to cases where the treatment enhances the S‐FUS[1–359] phenotype. Applying this approach, we show that a large fraction of DEG observed in the comparison of S‐FUS[1–359] and wild‐type samples have undergone the compensation in their expression levels (compensation coefficient between 40 and 100 for 60% of DEG), indicating that DF‐402 treatment substantially ameliorates the gene expression changes induced by FUS transgene expression in the spinal cord neurons (Figure 4B, Figure S3). Interestingly, the compensation applies to many genes upregulated in the presence of pathogenic FUS protein, resulting in the reversion of expression toward lower levels typical for expression of these genes in the spinal cord of wild‐type animals (lower cluster in Figure 4C, Figure S4A‐D). In contrast, genes that were downregulated in the spinal cord of transgenic mice showed less compensation (upper cluster in Figure 4C, Figure S4E). Genes from the middle cluster in Figure 4C showed no compensation or even a trend toward overcompensation (Figure S4F,G).

## 4 CONCLUSIONS

### 4.1 Multifactorial analysis of CatWalk testing results detects gait changes in FUS transgenic mice at early pre‐symptomatic stage

CatWalk is an effective combination of hardware and software in an experimental system designed for simultaneous analysis of multiple parameters of rodent gait.^53^, ^54^ It has been successfully used to assess changes in animal motor function in several transgenic models of neurodegenerative diseases.^55^, ^56^, ^57^, ^58^ Here, this system was used for the first time to detect motor deficiency in transgenic mice modeling ALS‐FUS by neuronal expression of pathogenic truncated form of human FUS protein.^33^

Our analysis of parameters obtained by CatWalk testing of cohorts of transgenic and wild‐type littermate mice allowed detecting gait changes in S‐FUS[1–359] mice already at early pre‐symptomatic stage, that is, long before manifestation of obvious clinical signs of the disease. This paves way for developing a protocol for accurate prediction of the time of the disease onset in an individual S‐FUS[1–359] mouse at least a week in advance, which can be considered as an equivalent of the detection of very early ALS symptoms in human patients. Such protocol, which currently goes through final stages of testing in our laboratories, will permit normalizing start of a drug and placebo administration at pre‐symptomatic stage within a cohort of experimental and control S‐FUS[1–359] mice used for preclinical trials.

By comparing data obtained in longitudinal studies of the CatWalk performance of control and DF402‐treated S‐FUS[1–359] mice, we demonstrated that a multifactorial analysis of gait parameters used in this study is sufficiently robust to detect and monitor even mild effects of drugs or other therapeutic interventions from the early stage of motor neuron disease in this transgenic model. Therefore, the algorithm of the CatWalk analysis developed in this study is a powerful tool for accurate assessment of the development of motor dysfunction in mice.

### 4.2 DF402, a bioisostere of Dimebon, ameliorates pathology in FUS transgenic mice

We have found that chronic treatment with DF402, a bioisostere of Dimebon, an over‐the‐counter drug that demonstrated a mild ability to suppress pathological changes in several models of neurodegenerative diseases, slowed down the development and progression of the disease in S‐FUS[1–359] mice with a statistically significant prolongation effect observed on all three main parameters, namely time to the disease onset (+11%), disease duration (+24%), and animal lifespan (+13%). Although it is difficult to directly compare results of studies that used different experimental setups and endpoints, the efficacy of chronic administration of a low dose of DF402 via drinking water is comparable and possibly higher than the efficacy of a similar protocol of chronic Riluzole administration to S‐FUS[1–359] mice.^59^, ^60^, ^61^ It should be also noted that chronic administration of Dimebon showed a delayed onset of clinical signs and an increase in lifespan of SOD1(G93A) mouse model of ALS,^28^ whereas the efficacy of Riluzole in this model was negligible.^62^ Two other bioisosteres of Dimebon, DF302 and DF312, have also been found capable to ameliorate disease parameters but not to prevent the development and progression of neuropathology in mouse models.^9^, ^12^ Taken together, these results suggest that these structures can be considered as promising lead compounds for further optimization to obtain novel medicines that might be used in combination with other drugs or therapeutic approaches to achieve efficient treatment of ALS patients.

### 4.3 Gene expression changes take place in the spinal cord of S‐FUS[1–359] mice already at early pre‐symptomatic stage

Two key mechanisms are involved in molecular pathogenesis of ALS‐FUS, namely impairments in RNA metabolism and pathological aggregation of FUS. Because C‐terminally truncated FUS 1–359 protein lacks major RNA‐binding domains,^63^, ^64^, ^65^, ^66^ its expression in the nervous system of transgenic mice cannot have direct effect on RNA metabolism. Therefore, S‐FUS[1–359] mice represent a model of pathology triggered solely by FUS aggregation and changes in the nervous system transcriptome of these mice reflect the development of FUSopathy‐induced pathology.

We found that changes in expression pattern for a limited number of genes in the spinal cord of S‐FUS[1–359] mice compared to wild‐type littermates happen long before transgenic mice develop first clinical signs of pathology. Because the expression of pathogenic FUS in S‐FUS[1–359] mice is neuron‐specific, we expected that at the pre‐symptomatic stage, that is, before any neuroinflammatory responses are developed, most of gene expression changes will be restricted to neurons. Our bioinformatic analysis demonstrated that at this stage, most DEG indeed belong to genes expressed in neurons.

### 4.4 Treatment with DF402 reverses changes of expression of many genes affected at the pre‐symptomatic stage of the pathology development in S‐FUS[1–359] mice

Bioinformatic analysis of RNA‐seq data also revealed that DF402 treatment affects spinal cord transcriptomes of S‐FUS[1–359] mice. It also demonstrated that genes differentially expressed in the spinal cord of S‐FUS[1–359] compared to wild‐type mice can be divided into two principal groups: those that undergo substantial reversion of their expression by DF402 treatment of S‐FUS[1–359] mice and those that display no or very little changes following DF402 treatment. As DF402 only slows down but cannot prevent the development of pathology, it is feasible to suggest that DEG belonging to the first group, DF402‐revertants, represent a group of genes and encoded proteins that have only marginal effect on the neurodegeneration in this model system. In contrast, genes belonging to the second group and their encoded proteins might play more substantial role in the progression of pathological changes from the pre‐symptomatic to symptomatic stage and therefore constitute more credible molecular targets for therapeutic intervention, at least in ALS‐FUS cases. Testing these suggestions will require further analysis of the observed mRNA changes, confirmation that they correlate with changes in the encoded protein levels, and detailed studies of the effects of modulation of each identified gene expression on the development of proteinopathy‐induced neurodegeneration in various experimental systems, all of which are beyond the scope of the current report.

## CONFLICT OF INTEREST

The authors have declared no competing interest.

## AUTHORS’ CONTRIBUTION

NN, SOB, and VLB conceived and supervised the study and analyzed the data. KC, APR, SF, TAI, EAL, and AYA carried out the experiments. KC, APR, SF, AVD, and MSK analyzed the data. VLB wrote the manuscript with contribution from all authors.

## Supporting information

Fig S1

Fig S2

Fig S3

Fig S4

Table S1

## Data Availability

The data that support the findings of this study are openly available in NCBI GEO database at https://www.ncbi.nlm.nih.gov/geo/, reference numbers GSE161680 and GSE130604.
