# Supplementary figures and images for: A bioisostere of Dimebon/Latrepirdine delays the onset and slows the progression of pathology in FUS transgenic mice
Source: CNS Neurosci Ther. 2021 Mar 23;27(7):765–75. doi: 10.1111/cns.13637 (PMC8193697; doi:10.1111/cns.13637)

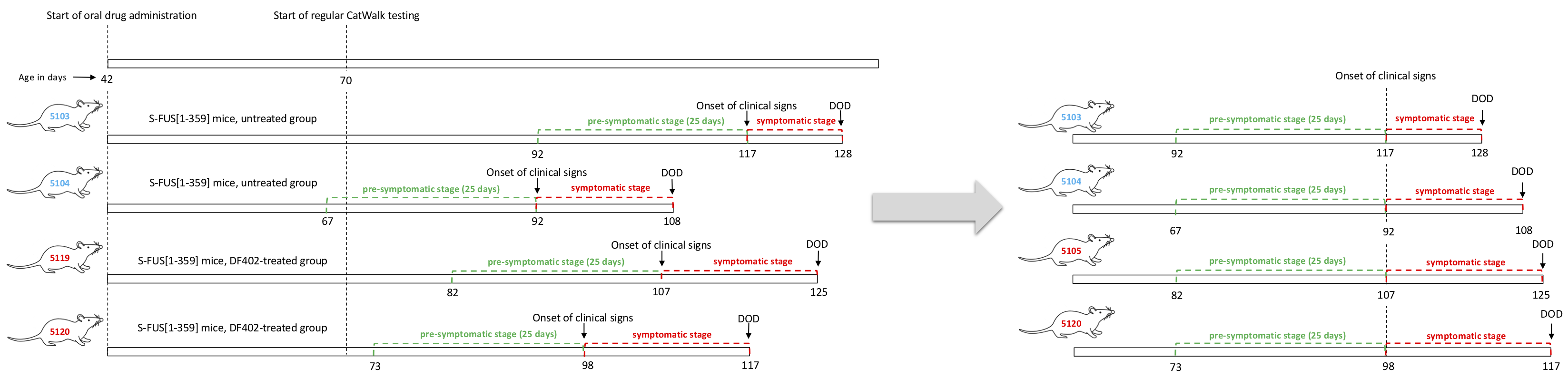

Supplement: Supplementary file 1 — Fig S1 [file CNS-27-765-s002.tif]

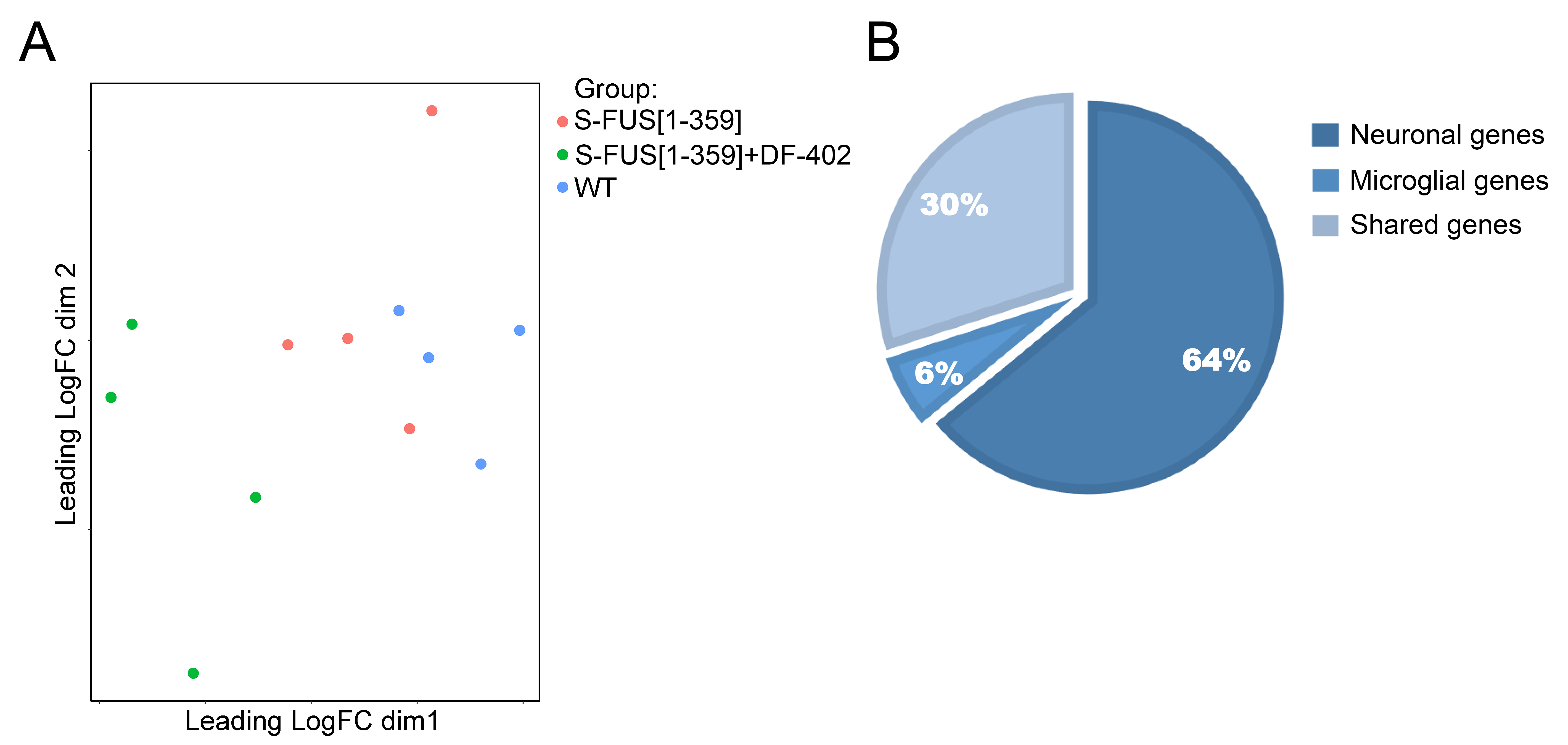

Supplement: Supplementary file 2 — Fig S2 [file CNS-27-765-s001.tif]

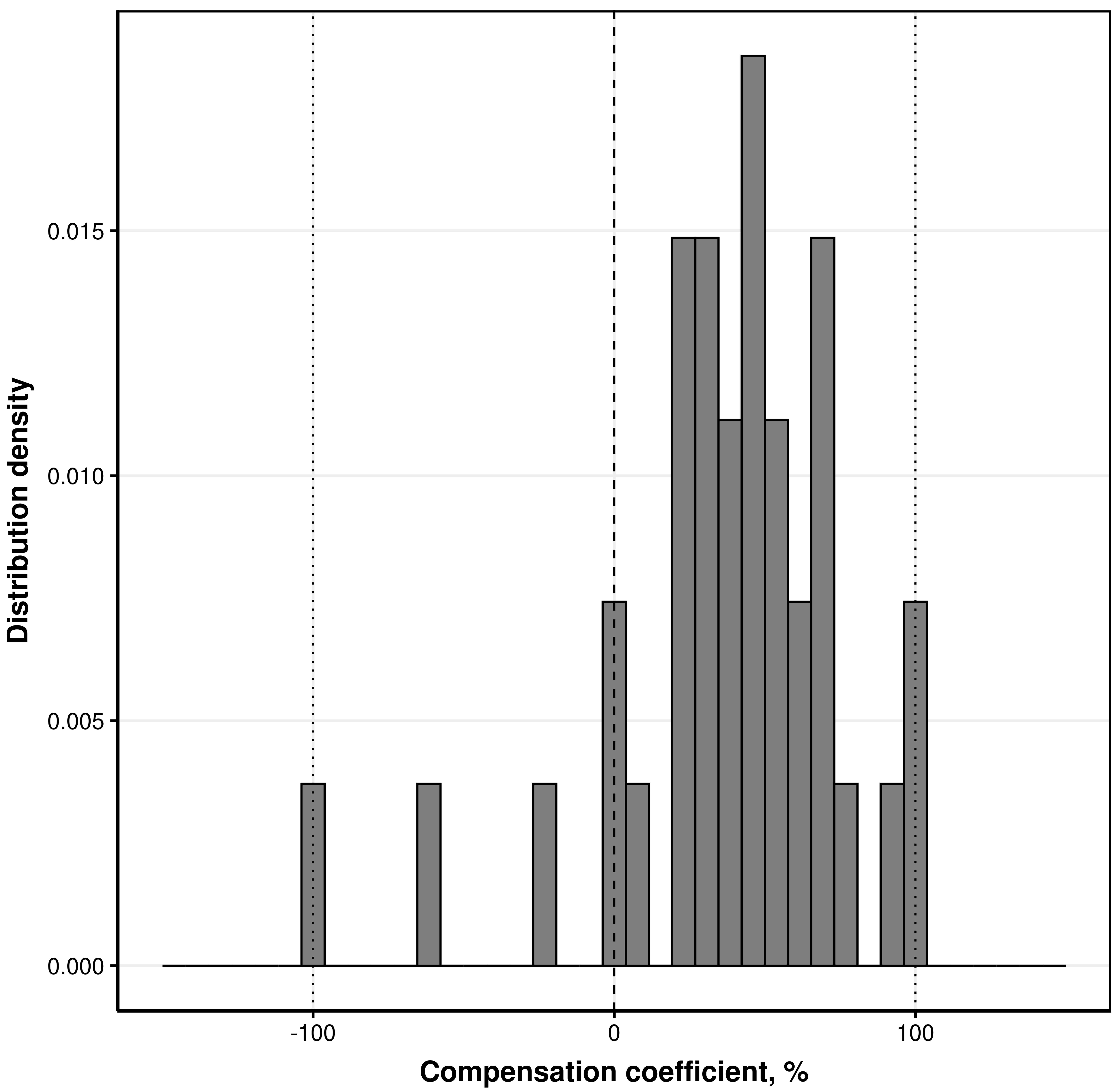

Supplement: Supplementary file 3 — Fig S3 [file CNS-27-765-s005.tiff]

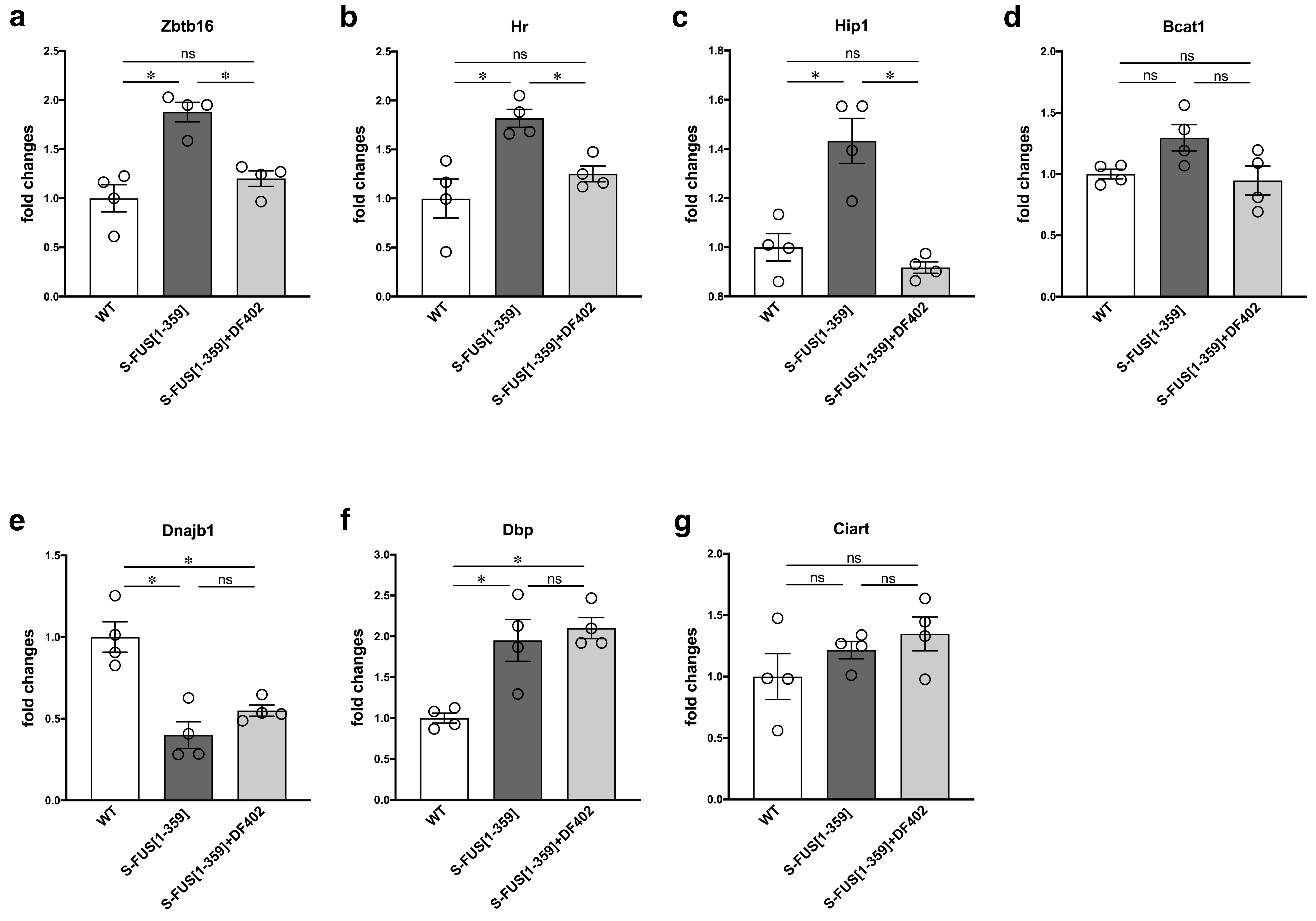

Supplement: Supplementary file 4 — Fig S4 [file CNS-27-765-s003.tiff]
